# Machine Learning‐Based Text Processing Reveals Research Trends in Heterotopic Ossification

**DOI:** 10.1155/ancp/9959615

**Published:** 2026-07-16

**Authors:** Yangbai Sun, Qinyuan Zhu, Chaoran Yu, Chunmeng Wang, Wangjun Yan, Chaoyin Jiang

**Affiliations:** ^1^ Department of Orthopedics, Shanghai Sixth People’s Hospital Affiliated to Shanghai Jiao Tong University School of Medicine, Shanghai, China, sjtu.edu.cn; ^2^ Department of Musculoskeletal Oncology, Bone and Soft Tissue Sarcomas Centre, Fudan University Shanghai Cancer Centre, Fudan University, Shanghai, China, fudan.edu.cn; ^3^ Department of Dermatology, Huashan Hospital, Fudan University, Shanghai, China, fudan.edu.cn; ^4^ Department of General Surgery, Shanghai Ninth People’s Hospital, Shanghai Jiao Tong University School of Medicine, Division for Shanghai Ninth People’s Hospital, China Hospital Development Institute, Shanghai Jiao Tong University, Shanghai, China, sjtu.edu.cn

**Keywords:** bibliometric analysis, heterotopic ossification, machine learning, Web of Science

## Abstract

**Background:**

Heterotopic ossification (HO) represents a highly active research field in pathological bone formation. Despite substantial advancements, a comprehensive understanding of its underlying mechanisms and clinical trajectory remains incomplete.

**Materials and Methods:**

A bibliometric and machine‐learning latent Dirichlet allocation (LDA) analysis was performed using data retrieved from the Web of Science Core Collection database.

**Results:**

A total of 4722 publications related to HO were identified. The most prolific countries included the USA, CHINA, GERMANY, the UK, JAPAN, SOUTH KOREA, ITALY, TURKEY, FRANCE, and CANADA . GERMANY demonstrated the highest citation strength, followed by CHINA . Aside from “heterotopic ossification,” other frequently occurring author keywords included “fibrodysplasia ossificans progressive,” “complications” and “total hip arthroplasty.” In keywords plus, besides HO, replacement, bone‐formation, and arthroplasty were the most frequently occurring terms. Institutional network analysis with subject‐specific clustering indicated that Shanghai Jiao Tong University was significantly enriched in radiology, nuclear medicine and medical imaging, while Wilderness Spine Serv specialized in surgical management. A developmental timeline plot of a network of most contributing authors also was visualized, along with the most influential references. Meanwhile, citation analysis indicated that Kaplan FS and Shore EM were the top‐cited authors. By LDA analysis, a total of 16 key topics were identified in this field with distinct period‐proportion visualization. One of the topics, cell bone express differentiation and formation has clearly dominated the last 10 years.

**Conclusion:**

This study constitutes the most extensive text processing analysis of HO to date, offering valuable insights and directions for future development.

## 1. Introduction

Heterotopic ossification (HO) is defined as the formation of bone in soft tissue where it does not normally exist, distinct from pathological calcification, such as that occurring in hypercalcemia associated with metastatic lesions [1]. Clinical manifestations may include fever, swelling, erythema, and a restrained joint motion range [2]. HO is widely regarded as a dysregulated tissue‐repair process and is listed among the most common complications of surgery or trauma. Its diverse clinical presentation has made it a subject of extensive investigation, with some lesions exhibiting significant morbidity, while others remain small and asymptomatic [1–3].

Pathogenesis of HO often initiates with the activation of inflammatory signaling pathways, such as those mediated by COX‐2 or retinoic acid receptor, as well as intramembranous or endochondral ossification mechanisms [3–6]. Genetic mutations also contribute significantly, with two main types: fibrodysplasia ossification progressive (FOP) and progressive osseous heteroplasia (POH). FOP is primarily associated with ACVR1 mutations in early childhood, while POH results from inactivation mutations in GNAS1 [7, 8].

Despite notable progress, the pathophysiology of HO remains distinct from oncologic progression or normal biological repair. Given the growing volume of publications on the topic, a bibliometric analysis is warranted to fully characterize the research trends and identify emerging future perspectives. This study included the largest number of publications and the most practical perspectives for clinical research. The main significance of this study is its contribution to the whole picture of research progress in HO and further insights on future perspectives. In fact, not only was a thematic map was established for theme evaluations but also a machine learning‐based language model was built for topic evaluations and evolvement. One of the topics, cell bone express differentiation and formation, has clearly dominated the last 10 years.

This study represents the most comprehensive bibliometric analysis of HO to date, encompassing 4722 publications ranging from 2000 to 2024. The USA is the leading productive country in this analysis, with 1649 publications, more than the combined productions from several other countries. Bibliometric analysis provides innovative and practical perspectives to navigate future development and clinical practice in HO. Over the last 20 years, this research community has generated a wealth of articles, case reports, systematic reviews, and perspectives that contribute to understanding the mechanistic challenges in HO pathogenesis [9–14]. The rapid growth of publications around the globe underscores the field’s dynamism and promise.

## 2. Materials and Methods

HO was searched in the Web of Science Core Collection database (https://clarivate.com/webofsciencegroup/solutions/web-of-science-core-collection) using the following search strategy:

#1, TOPIC (heterotopic); #2, TOPIC (ossification); #3, Publication time (January 1st, 2000 to February 1st, 2024); #4, Language (English). The final search results were retrieved based on “#1 AND #2 AND #3 AND #4,” and output for further data extraction (including a list of bibliometric variables such as author lists, countries, institutions, citations, references, publication times, journals, etc.) and interpretations based on the Bibliometrix packages and topic modeling algorithms of latent Dirichlet allocation (LDA) in R software (Version 4.0.2) and CiteSpace software (Version 6.3.1). Raw data cleaning and duplication deletion were processed based on the duplicatedMatching function and the restricted Damerau‐Levenshtein distance algorithm in R software. Meanwhile, to further extract insightful topics from textual data, a machine learning‐based LDA model in natural language processing was applied. By inputting a huge amount of documents, it sifts through and discerns recurring patterns of hub words, revealing potential semantic themes [15–21].

## 3. Results

**Figure 1:**
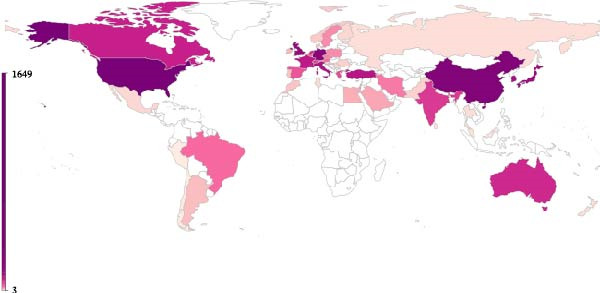
Global map of countries associated with publications in heterotopic ossification from 2000 to 2024.

**Figure 2:**
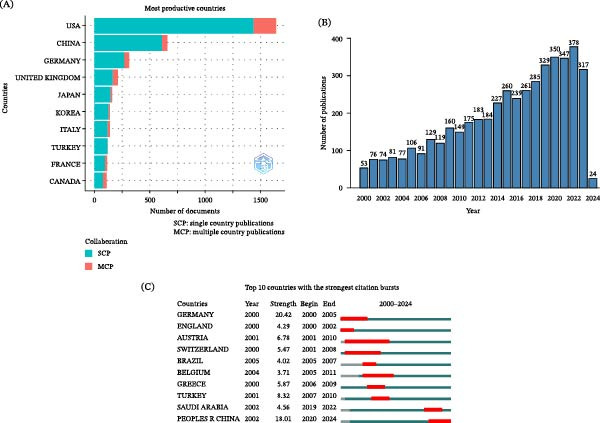
Publication of heterotopic ossification from 2000 to 2024. (A) Studies of heterotopic ossification from the top 10 countries; (B) annual publication of heterotopic ossification from 2000 to 2024; (C) citation analysis of publications in the top 10 countries.

Our search identified a total of 4722 publications relating to HO from the Web of Science. The leading countries with the most publications include the USA, CHINA, GERMANY, the UK, JAPAN, KOREA, ITALY, TURKEY, FRANCE, CANADA (Figures 1 and Figure 2A). Annual publication counts indicated a steady increase, peaking in 2022, reaching over 378 (Figure 2B, Supporting Information 1: Figure S1). Citation analysis revealed GERMANY as the leading country with the highest citation strength (strength = 20.42) and citation period between 2000 and 2005, followed by CHINA (strength = 18.01) and the citation period between 2020 and 2024 (Figure 2C). However, average article citation per year remained in steady decline since 2018 (Supporting Information 2: Figure S2).

**Figure 3:**
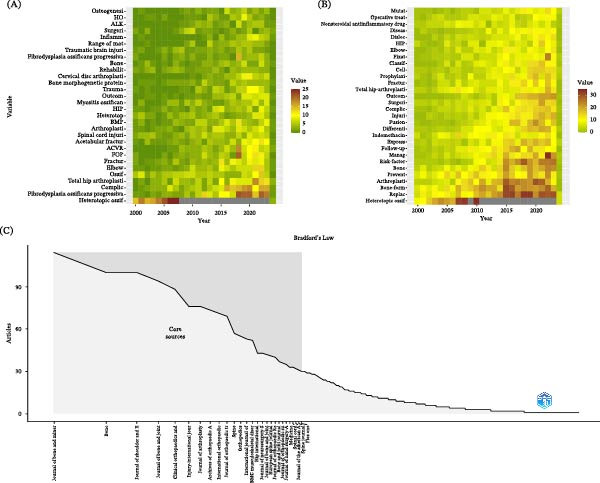
Annual occurrences of keywords of recruited studies from 2000 to 2024 and core sources presentation. (A) Annual occurrence of the top 30 author keywords; (B) annual occurrence of the top 30 keywords plus; (C) core sources presentation via Bradford’s law of publications in highlighted journals.

Next, keyword analysis revealed differences between author keywords and keywords plus (Figure 3). Besides “heterotopic ossification,” the most frequent author keywords were “fibrodysplasia ossificans progressive,” “complications,” and “total hip arthroplasty.” In keywords plus, besides “heterotopic ossification,” “replacement,” “bone‐formation” and “arthroplasty” were the three most frequently occurring terms (Figure 3A,B). Other rising keywords were “prevent,” “bone,” “risk factor,” “management,” “follow‐up,” “express,” and “indomethacin.” A keywords occurrence network is also visualized (Supporting Information 3: Figure S3). According to Bradford’s law, the most contributing journals were the *Journal of Bone and Mineral Research*, *Bone*, *Journal of Shoulder and Elbow Surgery*, *Journal of Bone and Joint Surgery*, and *Clinical Orthopaedics and Related Research* (Figure 3C).

**Figure 4:**
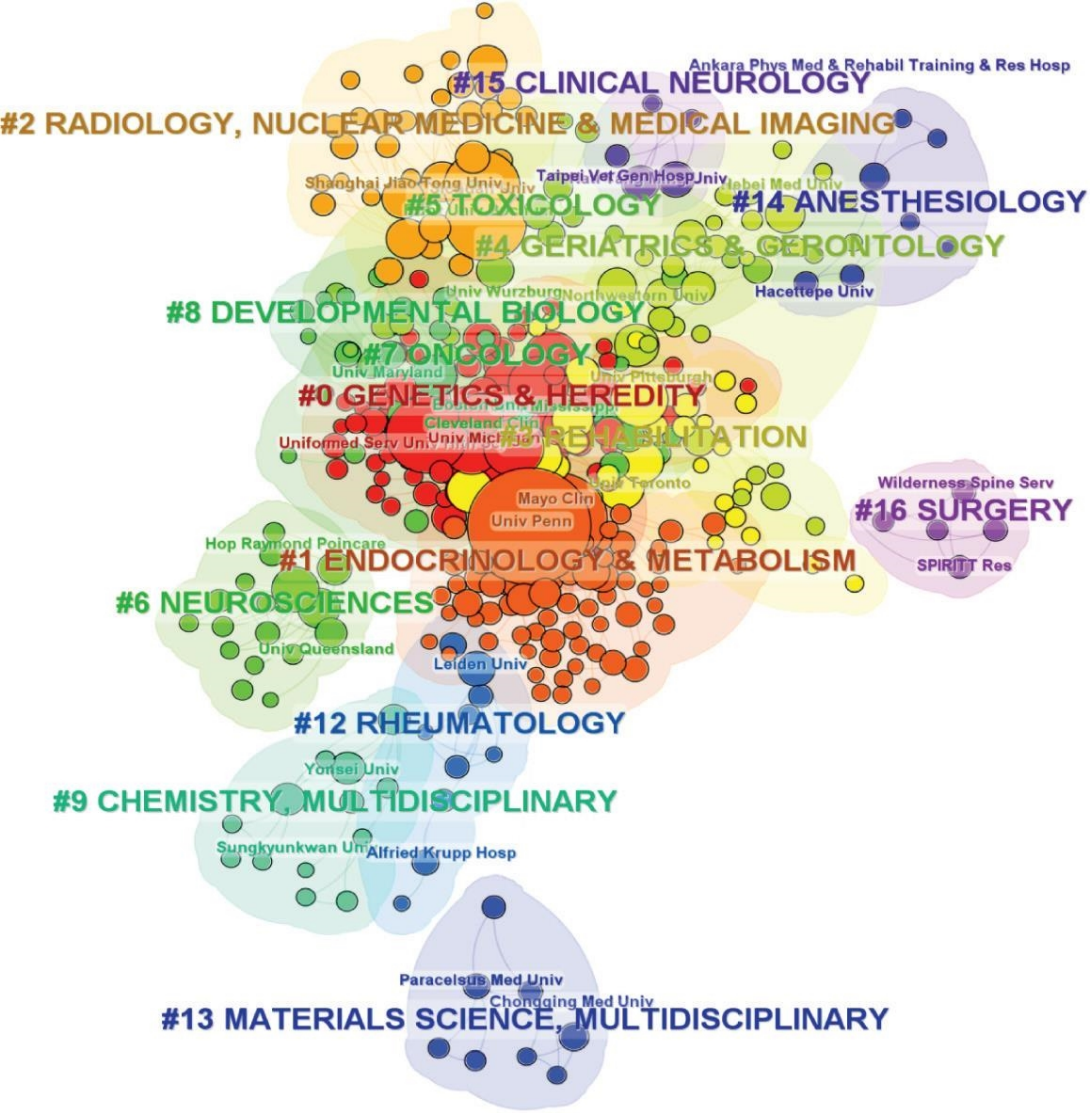
Network analysis of top institutions and subject classification in heterotopic ossification around the world. The network of institutions was colored based on each research subject classification.

Institutional network analysis with subject‐specific trends showed that Shanghai Jiao Tong University was significantly prominent in radiology, nuclear medicine, and medical imaging, while Wilderness Spine Serv specialized in HO surgery (Figure 4). Our results highlight the diversity of author institutions, topics, interactions, and research themes within HO. Paracelsus Med Univ and Chongqing Med Univ were associated with materials science and multidisciplinary. Leiden Univ and Alfried Krupp Hosp specialized in rheumatology. Yonsei Univ and Sungkyunkwan Univ were in chemistry and multidisciplinary. Shanghai Jiao Tong University was significantly prominent in radiology, nuclear medicine, and medical imaging. Wilderness Spine Serv was in surgery for HO. It offered an insightful clue and potential avenues for future collaboration opportunities.

**Figure 5:**
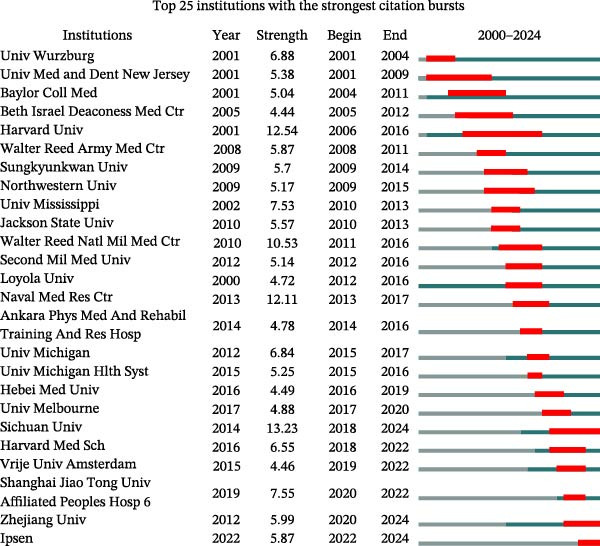
Citation analysis of the top 25 institutions associated with heterotopic ossification from 2000 to 2024.

**Figure 6:**
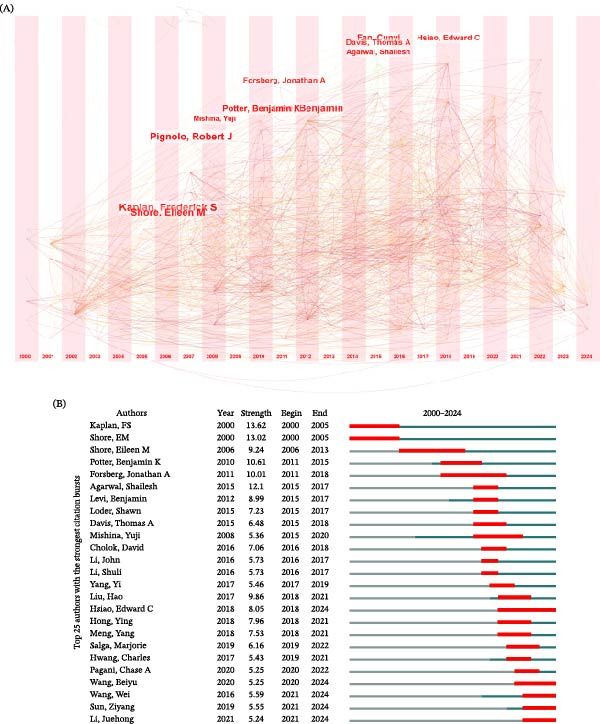
Citation analysis of authors associated with heterotopic ossification. (A) Network analysis of authors with the most publications from 2000 to 2024; (B) the top 25 authors with the strongest citations.

**Figure 7:**
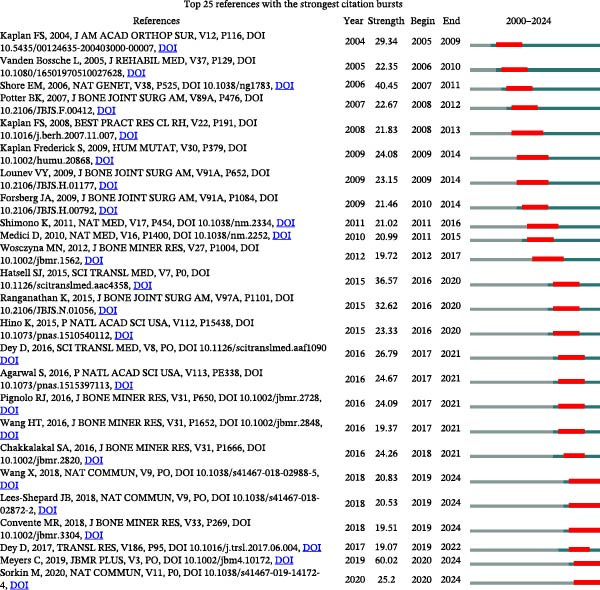
Citation analysis of the top 25 references associated with heterotopic ossification from 2000 to 2024.

**Figure 8:**
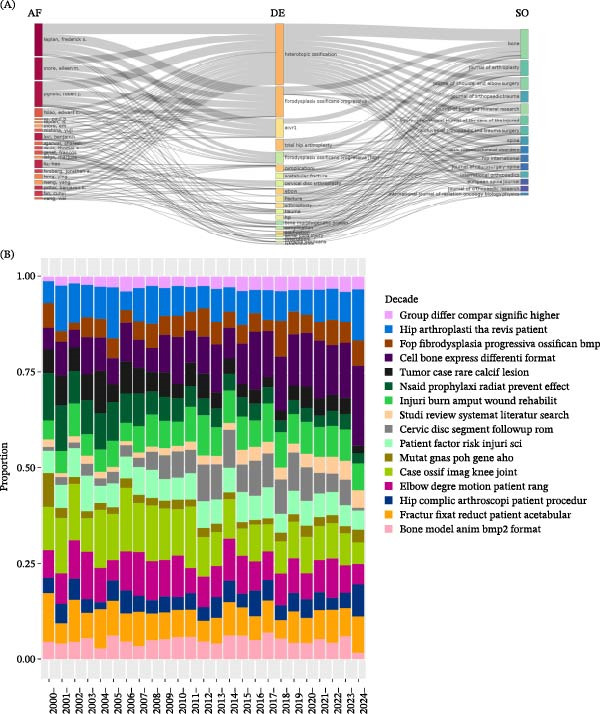
Text mining revealed distinct features in authors, keywords, publications, and topic modeling. (A) Sankey visualization of the association between authors, keywords, and published journals. AF: author; DE: author keywords; SO: journals; (B) topic modeling and topic proportions alteration over a decade; a total of 16 topics were marked by various colors.

Among the total of 25 institutions with the strongest citation burst, Sichuan Univ (strength = 13.23), Naval Med Res Ctr (strength = 12.11), and Harvard Univ (strength = 12.54) were the top three institutions. Noticeably, Harvard Univ also exhibited the longest citation burst period from 2006 to 2016 (Figure 5). Next, to characterize the contributions of authors, a timeline plot of a network of the most contributing authors was visualized (Figure 6A). Meanwhile, citation analysis indicated that Kaplan FS and Shore EM were the top‐cited authors (Figure 6B). The most influential references with the strongest citation bursts were identified (Figure 7). In the end, to characterize the association among authors, keywords, and journals, a Sankey diagram illustrated relationships among authors, keywords, and journals, reaffirming the prominence of Kaplan FS, Shore EM, and Pignolo RJ, and “heterotopic ossification,” “fibrodysplasia ossificans progressive,” “acvr1,” and “total hip arthroplasty” in top keywords, and *Bone*, *Journal of Arthroplasty*, and *Journals of Shoulder and Elbow Surgery* in the top three relevant journals (Figure 8A). To further characterize the theme development, a thematic map by development degree‐relevance degree analysis was produced. Themes such as fusion and expression showed high density but low centrality, indicating a comparably high developmental status but low relevance. Meanwhile, themes such as replacement and HO, on the contrary, showed high centrality but low developmental degree (Supporting Information 4: Figure S4). In order to offer more insights, machine learning‐based LDA models were introduced, a total of 16 topics were identified. A topic visualization was established for a comprehensive overview of the changing proportions of each topic across different time periods (Figure 8B, Supporting Information 5: Figure S5). One of the topics, cell bone express differentiation and formation, has clearly dominated the last 10 years.

## 4. Discussion

This study represents the most comprehensive bibliometric analysis of HO to date, encompassing 4722 publications ranging from 2000 to 2024. The USA is the leading productive country in this analysis, with 1649 publications, more than the combined productions from several other countries. Bibliometric analysis provides innovative and practical perspectives to navigate future development and clinical practice in HO. Over the last 20 years, this research community has generated a wealth of articles, case reports, systematic reviews, and perspectives that contribute to understanding the mechanistic challenges in HO pathogenesis [9–14]. The rapid growth of publications around the globe underscores the field’s dynamism and promise. Similar reports of HO remain sparse. In 2013, Liu et al. [22] studied the mesenchymal stem cells in HO in ankylosing spondylitis, with a total of 127 publications. In this study, China showed the leading publications. Most recent studies were focused on “osteogenic differentiation,” “gene expression,” and “inflammation.” Mesenchymal stem cells are growing to be a relatively new topic, with intensified research into abnormalities of the cells and the inflammatory microenvironment. Similarly, in our results, “inflammation” was listed as one of the rising author keywords, while “nonsteroidal anti‐inflammatory drug” and “indomethacin” were among keywords plus. In addition, we also expanded the study area of HO and the research time, yielding a total of 4722 publications, and further introduced a machine learning‐based language processing algorithm, the LDA model, into topic identification. A total of 16 relevant topics were visualized proportionally.

Notably, FOP and ACVR1 have attracted increasing research focus since 2018 [13]. Most of the FOP patients showed the same ACVR1 mutation, R206H [23–25]. Nonetheless, the BMP type I receptor was one of the additional mutations associated with ACVR1 [26]. Those mutations increase the BMP‐pSmad1/5/8 signaling pathway [27]. Three ACVR1 protein kinase domains, including G328E, G238W, and G356D, facilitate the responsiveness of the BMP ligand [28]. It is reasonably presumed that functional analysis of such mutations and genetic disorders will remain a central focus in the coming years.

Limitations remain in this study. Incomplete searches in this field and research community are highly sensitive to search strategies and databases. Meanwhile, more unpublished negative results or conference results are also of potential value to text mining. Mainly, there are also three more limitations. The reason why WoS was selected is mainly due to the high quality of articles indexed therein. WoS of Clarivate is commonly known as the highest reputed database with reliable citations and trusted quality control protocols, making it the primary choice for bibliometric analysis. However, other alternative databases, such as Scopus, are also of great value to broader resources.

Admittedly, self‐citations and publication bias may be some confounding factors for conclusions. However, WoS has initiated several annual quality checks and citation check processes to ensure one of the key misconducts, self‐citations, is properly controlled. That is also one of the key reasons we prefer WoS for citation analysis. However, English articles alone in this study may be biased to final results.

Initially, we also worried that newer studies such as those published in 2024 may be cited less frequently than previous publications. However, with the help of language processing, LDA models, and text processing, we screened all the texts and identified a total of 16 key topics, regardless of publication times, and presented them with an insightful heatmap for time‐proportion changes. Of note, a distinct increasing proportion has been noticed in some topics, which in fact indicate a sheer increment of citation and discussion (Figure 8B). This has helped us partially circumvent the disadvantages. However, for citation‐based metrics, there may be potential bias in the conclusions. Normalization of citations and reduction of self‐citation counts should be introduced for future metric comparison.

## 5. Conclusion

This study constitutes the most extensive bibliometric analysis of HO to date, offering valuable insights and directions for future development, with a topic visualization established for a comprehensive overview.

## Author Contributions

Yangbai Sun, Qinyuan Zhu, Chaoran Yu, Chunmeng Wang, and Wangjun Yan carried out data analysis, participated in study design, and data collection. Yangbai Sun, Qinyuan Zhu, Chaoran Yu, Chunmeng Wang, Wangjun Yan, and Chaoyin Jiang drafted the manuscript.

## Funding

This research was funded by the National Natural Science Foundation of China (Grants 82172415 and 82003351), Shanghai Municipal Health Commission Research Project (Grant 20194Y0242), Project of Hospital Management from Shanghai Ninth People’s Hospital, Shanghai Jiao Tong University School of Medicine (Grant YGB202412), Shanghai Municipal Health Commission 2025 Annual Commissioned Research Project on Health Legislation (Grant 6), China Hospital Development Institute, Shanghai Jiao Tong University 2026 Annual Decision‐Making Consultation Project (Grant CHDI‐2026‐Z‐030), and Fundamental Research Program Funding of Ninth People’s Hospital Affiliated to Shanghai Jiao Tong University School of Medicine (Grant JYZZ189).

## Disclosure

All authors read and approved the final manuscript.

## Ethics Statement

This article does not contain any studies with human participants or animals performed by any of the authors.

## Consent

No informed consent was needed in the study.

## Conflicts of Interest

The authors declare no conflicts of interest.

## Supporting Information

Additional supporting information can be found online in the Supporting Information section.

## Supporting information


**Supporting Information 1** Figure S1: Annual publications of articles from 2000 to 2024.

**Supporting Information 2** Figure S2: Annual publications of average article citations from 2000 to 2024.

**Supporting Information 3** Figure S3: Core keywords co‐occurrence networks. Subgroups were clustered and marked by various colors.

**Supporting Information 4** Figure S4: Thematic evolution analysis map in HO. It consisted of four quadrants, including themes in the top right area with well‐developed, top left area with comparable low relevance, bottom left area with little marginal value, and bottom right area with potential transdisciplinary value.

**Supporting Information 5** Figure S5: Identification of optimal number of topics. A total of four metrics were employed for assessment of optimal topic number, including CaoJuan2009, Deveaud2014, Griffiths2004, and Arun2010.

## Data Availability

The data that support the findings of this study are available from the corresponding author upon reasonable request.
