# Supplementary figures and images for: Machine Learning‐Based Text Processing Reveals Research Trends in Heterotopic Ossification
Source: Anal Cell Pathol (Amst). 2026 Jul 16;2026:9959615. doi: 10.1155/ancp/9959615 (PMC13373702; doi:10.1155/ancp/9959615)

# Annual Scientific Production

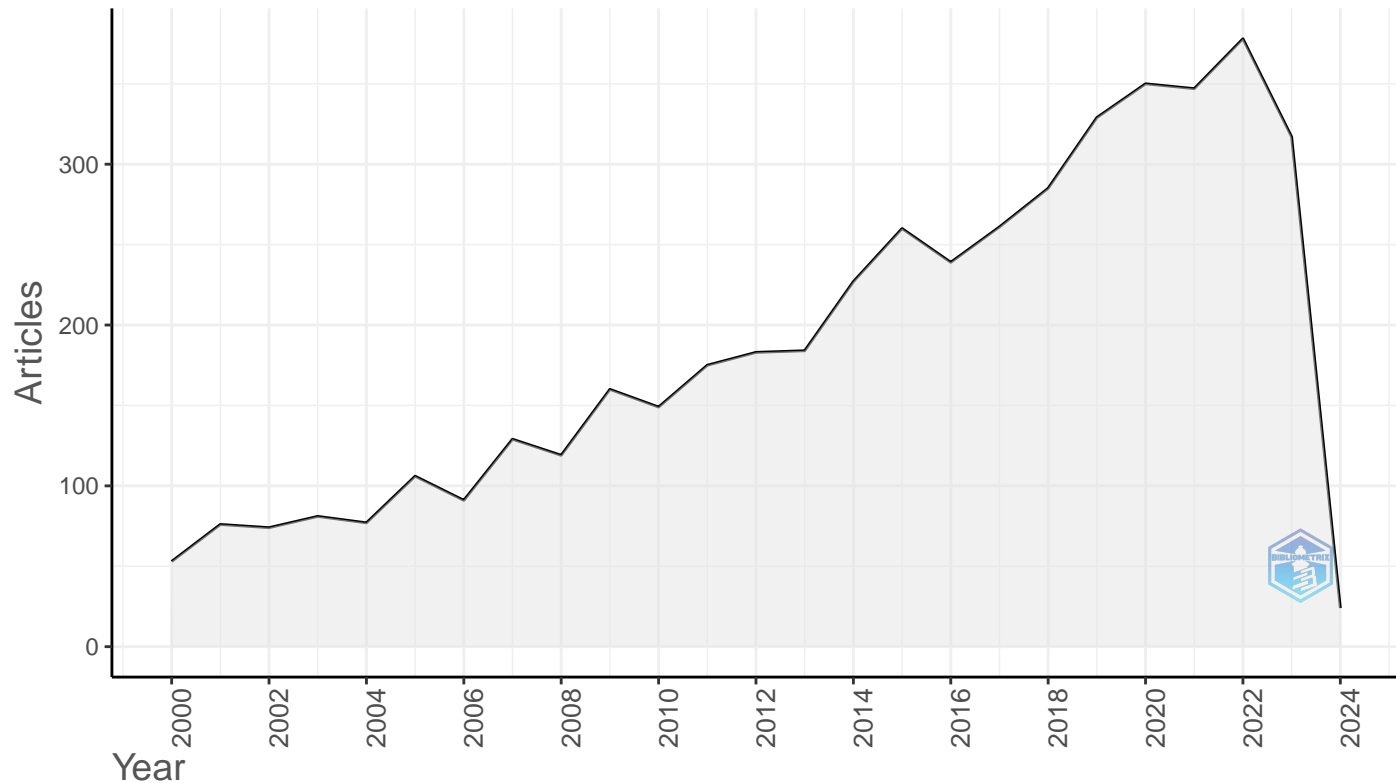

Supplement: Supplementary file 1 — Supporting Information 1 Figure S1: Annual publications of articles from 2000 to 2024. [file ANCP-2026-9959615-s002.pdf]

# Average Article Citations per Year

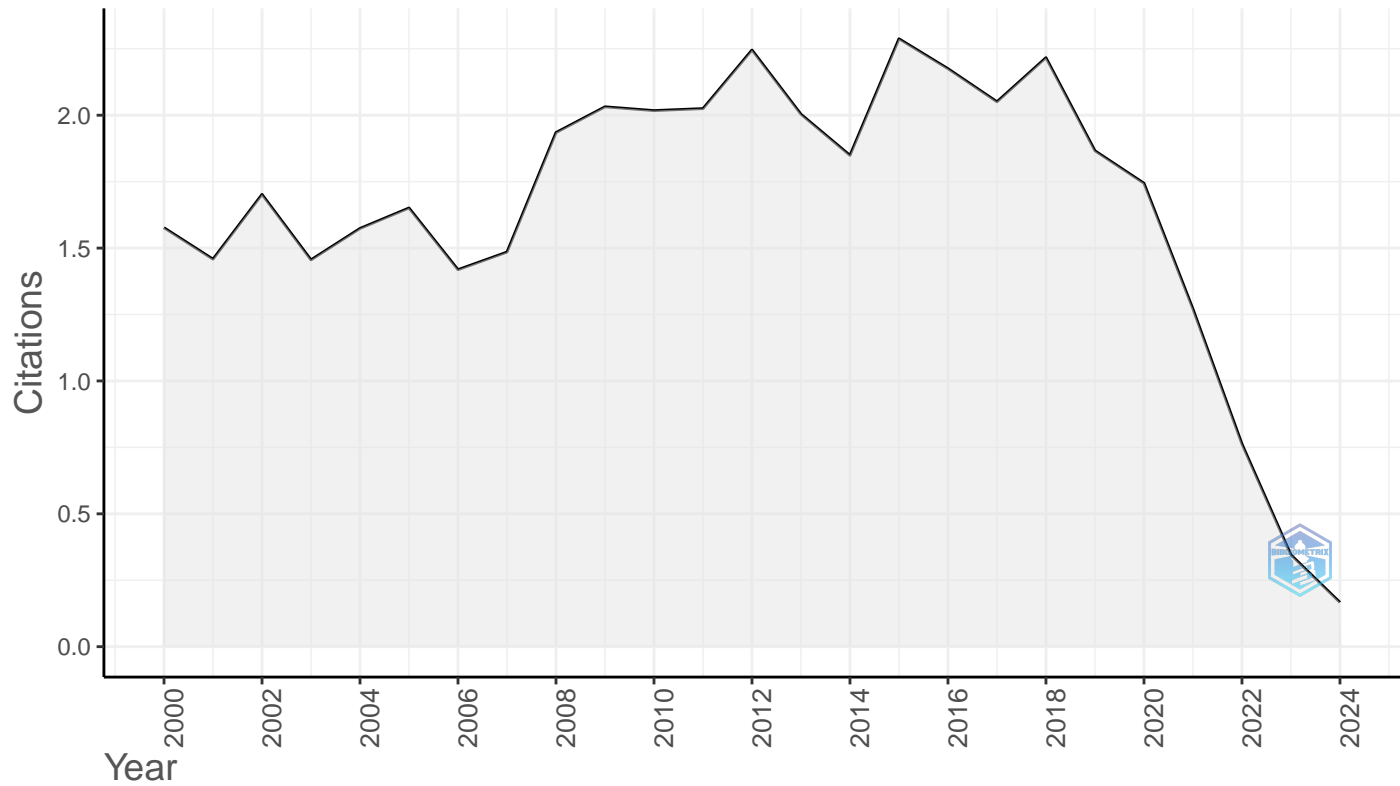

Supplement: Supplementary file 2 — Supporting Information 2 Figure S2: Annual publications of average article citations from 2000 to 2024. [file ANCP-2026-9959615-s001.pdf]

Keyword Co-occurrences

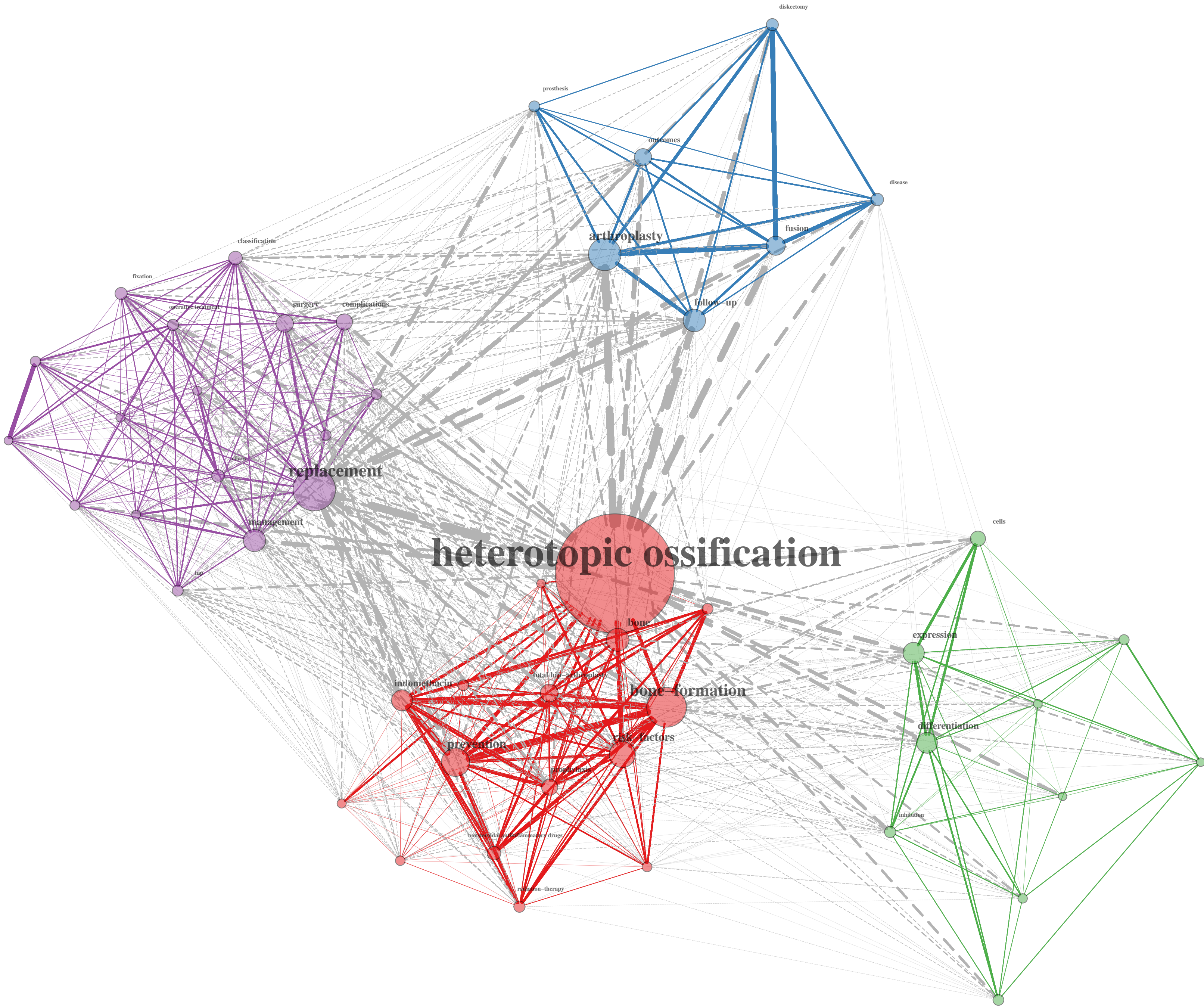

Supplement: Supplementary file 3 — Supporting Information 3 Figure S3: Core keywords co‐occurrence networks. Subgroups were clustered and marked by various colors. [file ANCP-2026-9959615-s005.pdf]

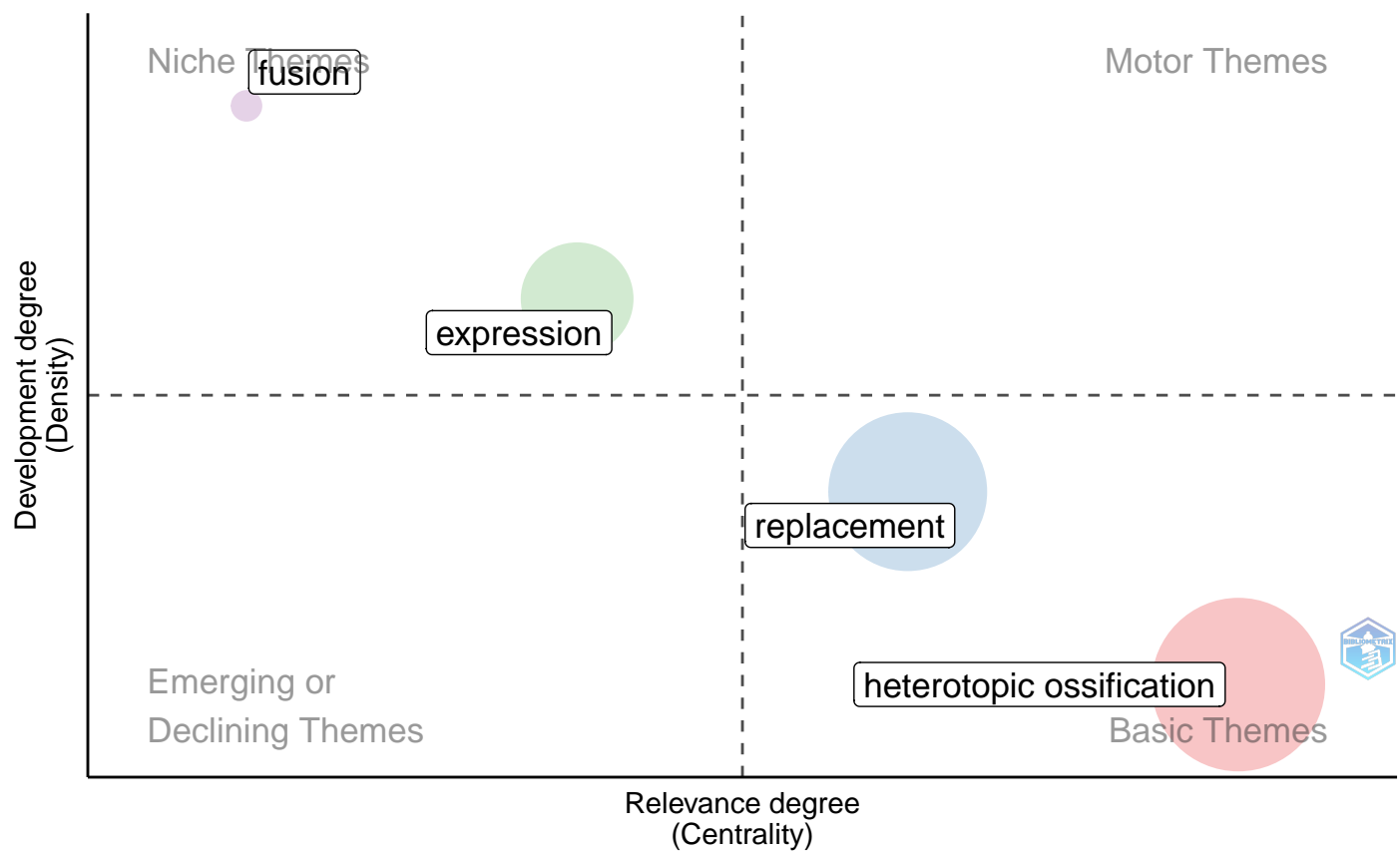

Supplement: Supplementary file 4 — Supporting Information 4 Figure S4: Thematic evolution analysis map in HO. It consisted of four quadrants, including themes in the top right area with well‐developed, top left area with comparable low relevance, bottom left area with little marginal value, and bottom right area with potential transdisciplinary value. [file ANCP-2026-9959615-s004.pdf]

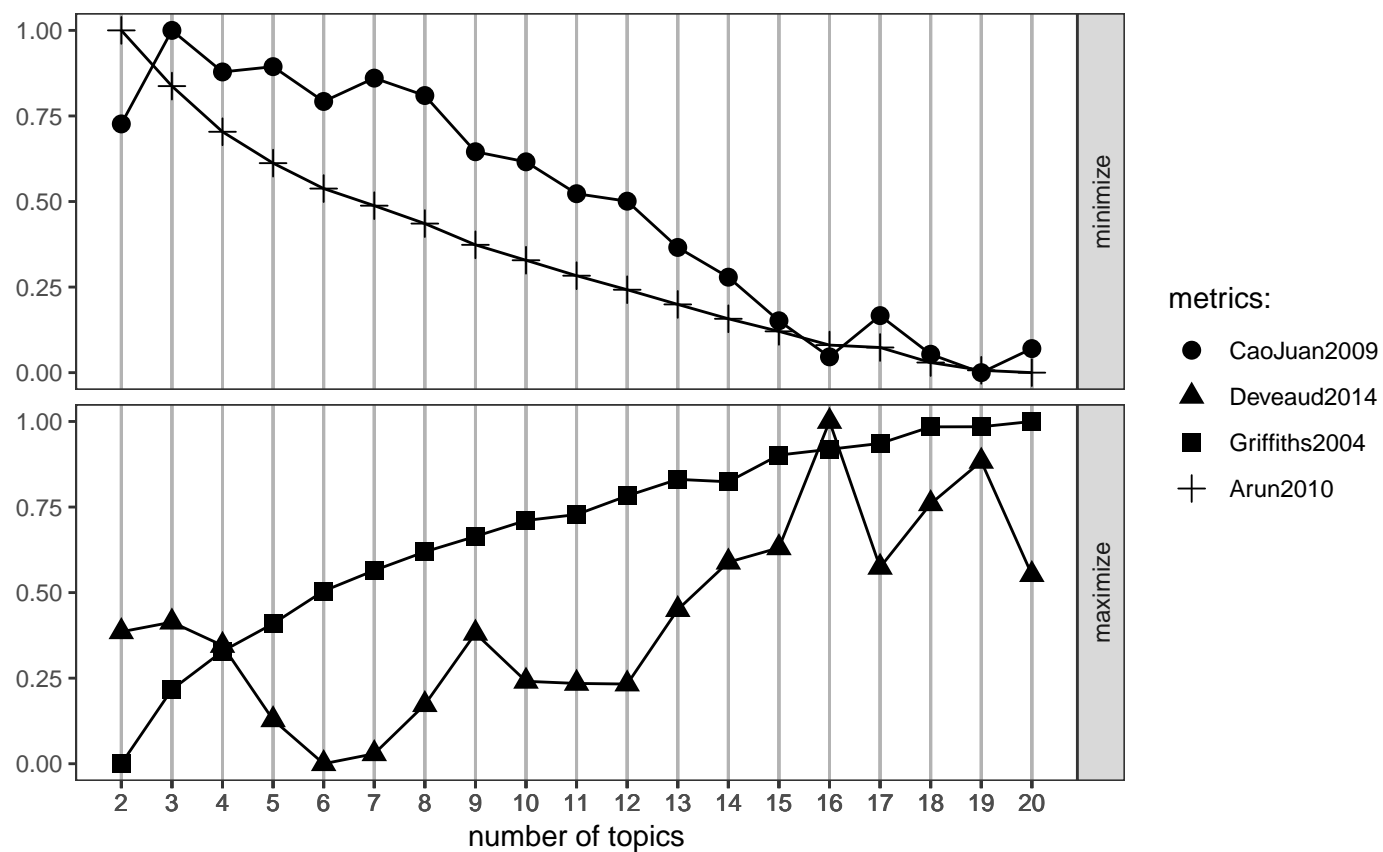

Supplement: Supplementary file 5 — Supporting Information 5 Figure S5: Identification of optimal number of topics. A total of four metrics were employed for assessment of optimal topic number, including CaoJuan2009, Deveaud2014, Griffiths2004, and Arun2010. [file ANCP-2026-9959615-s003.pdf]
